# Errors in Converting Principles to Protocols: Where the Bioethics of U.S. Covid‐19 Vaccine Allocation Went Wrong

**DOI:** 10.1002/hast.1416

**Published:** 2022-10-13

**Authors:** William F. Parker, Govind Persad, Monica E. Peek

**Keywords:** allocation, Covid‐19, public health, vaccines, scarce health care resources, bioethics

## Abstract

*For much of 2021, allocating the scarce supply of Covid‐19 vaccines was the world's most pressing bioethical challenge, and similar challenges may recur for novel therapies and future vaccines. In the United States, the Centers for Disease Control and Prevention's Advisory Committee on Immunization Practices (ACIP) identified three fundamental ethical principles to guide the process: maximize benefits, promote justice, and mitigate health inequities. We argue that critical components of the recommended protocol were internally inconsistent with these principles. Specifically, the ACIP violated its principles by recommending overly broad health care worker priority in phase 1a, using being at least seventy‐five years of age as the only criterion to identify individuals at high risk of death from Covid‐19 during phase 1b, failing to recommend place‐based vaccine distribution, and implicitly endorsing first‐come, first‐served allocation. More rigorous empirical work and the development of a complete ethical framework that recognizes trade‐offs between principles may have prevented these mistakes and saved lives*.

## Essay

After reviewing the proposed ethical principles for Covid‐19 vaccine allocation from the National Academy of Sciences, Engineering, and Medicine,^1^ the World Health Organization,^2^ and others,^3^ the U.S. Centers for Disease Control and Prevention's (CDC's) Advisory Committee on Immunization Practices supported three foundational principles for such allocation: maximize benefits and minimize harms, promote justice, and mitigate health inequities (see table 1).^4^ While not completely identical, the ACIP's principles substantially overlap the WHO and NASEM principles. We accept the ACIP's principles as guideposts for Covid‐19 vaccine allocation, but we believe that the allocation recommendations the ACIP made based on these principles are flawed (see table 2).^5^ This process of translating principles to protocols is essential, representing the conversion of abstract ethical concepts into practical rationing mechanisms. We describe four major breakdowns of this vital process and highlight errors of both omission (where the ACIP failed to make recommendations consistent with the set of chosen principles) and commission (where the ACIP's recommendations contradicted their stated ethical foundation).

**Table 1:** ACIP Ethical Principles for Allocation of Covid‐19 Vaccines^1^

*Principle* ^2^	*Dimensions*
Maximize benefits and minimize harms	Reduce SARS‐CoV‐2 infections and Covid‐19‐associated morbidity and mortality. Preserve services essential to the Covid‐19 response. Maintain overall societal functioning.
Promote justice	Protect and advance equal opportunity for all persons to enjoy the maximal health and well‐being possible. All persons have fundamental value and dignity.
Mitigate health inequities	Social position or other socially determined circumstances should not disadvantage a person's opportunity to attain full health potential. Reduce existing disparities. Do not create new disparities.

1 This table is adapted from N. McClung, “The Advisory Committee on Immunization Practices' Ethical Principles for Allocating Initial Supplies of COVID‐19 Vaccine—United States, 2020,” *Morbidity and Mortality Weekly Report* 69 (2020): 1782‐86.

2 The ACIP also supported the procedural principle of transparency.

**Table 2:** ACIP Final Recommended Phases for the Initial Covid‐19 Vaccine Allocation^1^

*Phase*	*Prioritized groups*
1a	All health care personnel Long‐term care facility residents
1b	Persons aged ≥ 75 Frontline essential workers
1c	Persons aged 65‐74 years Persons aged 16‐64 years with high‐risk medical conditions Other essential workers

1 Information in this table comes from K. Dooling, “The Advisory Committee on Immunization Practices' Interim Recommendation for Allocating Initial Supplies of COVID‐19 Vaccine—United States, 2020,” *Morbidity and Mortality Weekly Report* 69 (2020): doi:10.15585/mmwr.mm6949e1, and K. Dooling, “The Advisory Committee on Immunization Practices' Updated Interim Recommendation for Allocation of COVID‐19 Vaccine—United States, December 2020,” *Morbidity and Mortality Weekly Report* 69 (2021): doi:10.15585/mmwr. mm695152e2.

The ACIP comprises a group of “medical and public health experts who develop recommendations on the use of vaccines in the civilian population of the United States” that, if adopted by the CDC director, are published as official recommendations of CDC and the Department of Health and Human Services.^6^ While the ACIP's allocation guidance was not legally enforced, many states and cities based their allocation protocols heavily on its recommendations.^7^

## Overly Broad Definition of “Health Care Personnel” in Phase 1a

The first ethical misstep of the ACIP was an act of commission: it defined “health care personnel” so broadly as to leave many high‐risk persons in the community unvaccinated while vaccine doses went unused for months. When deciding who in the United States should have first access to the Covid‐19 vaccines, the ACIP assigned two groups equal priority: health care personnel (HCP) and residents of long‐term care facilities. These residents were a clear first choice to maximize benefits due to the combination of high infection risk in congregate living and the increased risk of severe postinfection outcomes because of advanced age and chronic medical conditions. From the perspective of promoting justice, residents of long‐term care facilities cannot protect themselves from infection by staff or visitors. Finally, prioritizing residents in these facilities may mitigate some racial health inequities: racial and ethnic minorities have access to lower‐quality long‐term care facilities and, prior to vaccine availability, suffered dramatically higher rates of nursing home Covid‐19 deaths than their White counterparts.^8^ However, some racial and ethnic minorities, such as Hispanic and Asian Americans, are substantially underrepresented in long‐term care facilities considering their share in the general U.S. population.^9^

In contrast, the ethical justification for the broad definition of HCP is less clear. The ACIP defined HCP as “all paid and unpaid persons serving in health care settings who have the potential for direct or indirect exposure to patients or infectious materials,” and health care organizations predictably operationalized this as anyone who works for a hospital or academic medical center.^10^ Totaling an estimated 21 million people, this expansive definition included basic science researchers and others far from the frontlines.^11^ While the vaccination of employees who were not in direct contact with patients was often portrayed in the media as a violation of the ACIP's recommendations,^12^ hospitals were simply using their allocated doses according to the expansive definition of HCP. Thus, the maximum‐benefits principle was violated in that many more vaccines would have been available for higher‐risk persons had there been a narrower definition of HCP; many people working for health care institutions who were not high risk or essential to the pandemic response were prioritized.

The argument for HCP priority rests primarily on the premise that “early protection of health care personnel is critical to preserve capacity to care for patients with Covid‐19 or other illnesses,”^13^ a phenomenon known as the multiplier effect. The ACIP did not include the ethical principle of reciprocity in its framework, but this principle likely played a silent and outsized role in establishing priority for HCP (and frontline essential workers). If the ACIP had either acknowledged or explicitly rejected the view that HCP should be prioritized not merely to preserve health system capacity but as reciprocity for the services they render to society, the committee would have improved the internal cohesion of the protocol and likely identified a narrower group of HCP as central to the pandemic response to vaccinate.

The ACIP asserted that broad health‐care‐worker priority was also supported by the increased occupational risk of Covid‐19. It is unclear whether this falls under the principle of promoting justice (if occupational exposure to Covid‐19 during the course of health care delivery is especially unjust) or the principle of maximizing benefits (because vaccinating highly exposed people prevents more harm). Either way, the assertion is dubious. While health care workers in the United States have a higher occupational risk of Covid‐19 infection, most had substantially higher access to free personal protective equipment than did other essential workers, reducing their relative risk of infection. Perhaps for this reason, rates of excess mortality from the virus did not significantly differ between HCP and frontline essential workers in other fields.^14^ Therefore, this strategy of prioritizing an overly broad pool of HCP for vaccination instead of only frontline workers with high occupational risk violated the justice principle. While the spillover multiplier benefits of vaccinated HCP to high‐risk patients and disadvantaged populations are likely positive, they do not outweigh the direct benefit of vaccinating these populations. The ACIP should have followed NASEM and recommended a narrow phase 1A priority for just those health care industry employees at high personal risk of severe outcomes if infected.

Finally, there are substantial racial and ethnic inequities in representation among higher‐income health care professionals (such as physicians and nurses).^15^ There was a hope that access for low‐wage health care workers, who are disproportionately racial or ethnic minorities, would create racial equity in vaccine utilization. However, Covid‐19 vaccine uptake was lower in these groups, consistent with existing literature about other vaccinations.^16^ Consequently, prioritization arrangements based on the ACIP's expansive definition of HCP created massive racial and ethnic inequity in vaccination rates. Therefore, the ACIP's protocol can be viewed as a failure of the disparities‐mitigation principle.

The ACIP protocol stands in stark contrast to the recommended jump‐start phase of first responders and high‐risk health care workers proposed by NASEM. Vaccinating a small group of workers essential to the pandemic response would have been consistent with the ACIP principles. Instead, hundreds of thousands of doses (over half the distributed supply) sat idle in freezers in January 2021.^17^ While there were many logistical reasons for the slow pace of vaccine uptake, reserving doses for the enormous HCP group defined by the ACIP's recommendations was a significant contributor.^18^ Ultimately, federal orders to expand eligibility to everyone sixty‐five years of age and over^19^ led to an immediate increase in Covid vaccinations.

## Recommending Seventy‐Five as the Age Minimum during Phase 1b

The second ethical error was using age as the single measure to determine eligibility for persons who were not frontline essential workers during phase 1b. In constructing phase 1b, the ACIP assigned equal priority to frontline essential workers and all persons seventy‐five years of age and older. The ACIP chose being over seventy‐five as a simple way to identify individuals at high medical risk, as about half of all U.S. Covid deaths prior to vaccine availability occurred in this age group.^20^ But this categorical age cutoff ignores the fact that the other half of deaths occurred in people *under* seventy‐five. Covid‐19 risk is a product of the probability of infection and death after infection. A wealthy seventy‐six‐year‐old able to live independently and shelter in place in their home is at substantially lower risk of Covid‐19 infection than a sixty‐six‐year‐old grandmother in a congregate living setting with multiple essential‐worker family members. Ignoring other medical risk factors also prioritizes individuals at lower risk over those at high medical risk. According to the protocol, a seventy‐three‐year‐old with diabetes and advanced congestive heart failure should receive lower priority for vaccination than a healthy seventy‐five‐year‐old.^21^ A one‐size‐fits‐all age cutoff fails to maximize benefits compared to approaches that account for place‐based risk and other medical risk factors.

One‐size‐fits‐all age thresholds were often defended on the basis of simplicity. But other factors, such as place‐based risk, could also be incorporated easily using the same types of identification documents used to confirm age.^22^ Occupational, medical, and other types of risk could also be incorporated at specific vaccination sites, such as hospitals vaccinating inpatients or employers organizing vaccine clinics. For example, teachers and patients in federally qualified health centers were prioritized for access in a way that cut across age thresholds. The same could have been done for other patients or employees. More broadly, if policy‐makers moved away from passive allocation approaches that require self‐reporting of eligibility criteria, other types of risk could be incorporated as part of active outreach. For instance, weighting of within‐phase vaccine lotteries could prioritize communities with high Covid burden or people with higher‐risk occupations or those with high‐risk conditions documented in their health records.

A strict age cutoff also violates the justice principle. By prioritizing only those who have been fortunate enough to reach seventy‐five years of age, this cutoff disproportionately disadvantages those who have not had the opportunity to live to seventy‐five and would be unlikely to even without the threat of Covid‐19.^23^ Because many types of disadvantage shorten life expectancy, people disadvantaged in other ways are less likely to live to seventy‐five. This inequity could justify vaccinating lower age ranges to ensure “equal opportunity for maximum health”^24^ over a life span.

Finally, the ACIP created significant structural inequities by race when they failed to account for the U.S. age distribution by race in this recommendation. The average age of a White person in the United States is fifty‐eight, more than twice the average age for racial and ethnic minorities.^25^ Ten percent of White Americans are at least seventy‐five years of age, compared to under 5 percent for minorities. By prioritizing Americans seventy‐five and over, the ACIP would have doubled the relative access of White people to vaccines. Compounding this further, the ACIP ignored wide racial disparities in Covid‐19 death risk at younger ages. Prior to vaccine availability, Native American, Black, and Hispanic people faced substantially higher Covid‐19 hospitalization and death rates for all age brackets under sixty‐five, such that members of these minority groups in the age bracket below sixty‐five were often at higher risk than White Americans sixty‐five and over. While the Covid‐19 mortality disparity also occurred (though was less stark) in adults over sixty‐five, the ACIP did not empirically demonstrate that this would counteract the fact that minority populations are half as likely to be over seventy‐five. It is unsurprising that, motivated partly by equity concerns, the federal government and most states ignored the seventy‐five‐plus recommendation, instead beginning phase 1b with sixty‐five years and older. This ACIP protocol was a clear violation of the disparity‐mitigation principle.

## Ignoring Place‐Based Risks and Geographic Variation

The third protocol error—one of omission—was the failure to consider place‐based risk and geographic variation in determining Covid‐19 vaccine distribution. Covid‐19 case rates, morbidity, and mortality have been unequally distributed across the United States since the start of the pandemic. In the first wave of the pandemic, death rates ranged from over eight hundred a day in New York to just one per day in Vermont.^26^ The geographic variation was even more pronounced over time, with some states being relatively spared from some waves, while other states were devastated. The lack of a firm directive from the CDC to allocate vaccines proportional to metrics of risk has had enormous consequences. For example, during the phased vaccine implementation, the United States faced a geographically concentrated fourth wave. The DHHS continued to allocate vaccines on a per capita basis (proportional to each state's population) rather than increasing allocation to the states with surging rates, an approach described by the DHHS secretary as “fair” despite its inconsistency with all the ACIP ethical principles.^27^

Within major cities, Covid‐19 was distributed along predicted lines of socioeconomic status and other social‐vulnerability measures, exacerbating long‐standing disparities in health care and health outcomes. According to multiple studies, racial and ethnic minority groups were more likely to be exposed to Covid‐19 and suffer severe outcomes like hospitalization and death at younger ages if infected.^28^ For example, 62.8 percent of the deaths during the first wave in Chicago were in Black patients, a disparity driven by higher social vulnerability and personal risk factors.^29^ The NASEM protocol recommended that states allocate 10 percent of their vaccine supply to high‐risk counties using the CDC's Social Vulnerability Index,^30^ as first proposed by Harald Schmidt in the *Hastings Center Report* in the spring of 2020.^31^ In response, two‐thirds of states considered geographical targeting in their vaccine allocation plans.^32^ The ACIP omitted this modest but important provision and did not recommend directing vaccines to the most vulnerable areas. And without the force of a formal ACIP recommendation, the practical implementation of these plans was lacking. Long‐standing structural inequities and shortcomings in the U.S. health care system contributed to a strong negative correlation between vaccine need and vaccination penetration at the county level.^33^

Further research confirms that this act of omission dramatically reduced the potential number of lives saved, particularly among vulnerable populations, violating both the principle of maximizing benefits and the principle of mitigating inequities by not allocating vaccines proportional to risk.^34^ In Chicago, 75 percent of deaths in the least vaccinated quartile of the city in the alpha and delta waves could have been prevented if that quartile had enjoyed the same vaccination rate as the highest vaccinated quartile.^35^ Vaccines have the potential to reduce harm from Covid‐19 where the virus is most prevalent. “Pouring the water where the fire is burning” should have been the guiding principle for geographic vaccine allocation. Arguments that it was “too late” to respond with more vaccines after a surge had begun were never substantiated by rigorous empirical studies.

Some may argue that asking individuals at low geographic, or place‐based, risk to wait for vaccines is inconsistent with the equal‐opportunity dimensions of the ACIP's “promoting justice” principle.^36^ If a community enjoys low viral risk because of strict adherence to nonpharmaceutical interventions, asking them to wait for vaccines might seem unfair. However, many nonpharmaceutical interventions (such as working remotely, having home delivery of food and other essential items, and living in homes that allow for single‐room isolation if needed) are far more feasible for wealthier people and less practical for or available to poorer people who face various challenges (such as densely populated and poorly ventilated buildings and low air quality). Geographically targeted allocation would satisfy the “equal opportunity for maximum health” dimension of promoting justice by actively accounting for the deep structural inequities documented by disadvantage indices like the CDC's Social Vulnerability Index.^37^

## No Within‐Phase Allocation Mechanism

The final ethical error was another act of omission. This was the failure to specify an ethical mechanism for within‐phase vaccine distribution. While the ACIP protocol defined allocation phases based on groups prioritized for vaccination due to increased risk of coronavirus infection or mortality, the allocation phases themselves were massive, ranging from 24 million to 129 million persons, with significant heterogeneity of within‐group risk (due to place‐based risk and other measures of social vulnerability, for example). The ACIP provided little direction for within‐phase allocation, aside from recommendations that local health authorities should “sub‐prioritize within an allocation phase if necessary.”^38^ Failing to directly engage with this sizeable gap in the allocation protocol created a vacuum that many health departments filled with first‐come, first‐served policies.

First‐come, first‐served approaches (rank ordering patients by waiting time) lead to equal opportunity for maximum health and well‐being only when it is possible for people with equivalent expected benefit to have equal access to the waiting list when they develop the need for the resource. For example, since requiring dialysis for end‐stage renal disease dramatically reduces health and well‐being, a system that ranks candidates for kidney transplantation by the date on which they need to start dialysis treatment would lead to equal waiting time suffering on dialysis. However, there is no analogous waiting time in a global pandemic. When each priority phase was activated, millions of people were instantly newly eligible for the vaccine.

A first‐come, first‐served approach prioritizes the well connected and well‐off, who have the luxury of time to wait in line, the financial resources to pay someone to wait for them, or the social capital to cut to the front of the line. While one could speculate that a first‐come, first‐served approach might select for those who are most desperate for a vaccine and further speculate that such people would benefit more than average, there is no evidence that this happened during Covid‐19 vaccine allocation. In reality, when combined with online‐only sign‐up systems, first‐come, first‐served vaccine allocation exacerbated health disparities (a process described as “digital redlining”), violating the principle of mitigating inequity.^39^

The ACIP should have strongly recommended randomized lottery systems to allocate vaccines within phases.^40^ Paired with autoenrollment of whole populations and proactive, culturally appropriate outreach, this would have dramatically improved the alignment of the ACIP protocol with its principles. Instead of passive approaches that left computer‐literate senior citizens struggling to refresh their browsers to get appointment slots, while those without technology access were unable to access vaccines at all, people could and should have received emails, text messages, phone calls, and door‐to‐door visits enabling them to sign up when their random number was selected. By neglecting to address how to decide between millions of people within each phase, the ACIP de facto prioritized people with the highest socioeconomic status (with the lowest risk of infection and mortality), who had the most available time to devote to the task of obtaining first‐come, first‐served vaccines, rather than those of the highest need, thereby violating the ethical principle of maximizing benefit.

## Why Protocol Development Went Wrong

There are consistent themes in the mistakes the ACIP made during protocol development. First, they did not engage empirical data properly when translating their ethics framework into recommendations. Rather than relying on intuitive speculation for many prioritization decisions (such as the use of age seventy‐five as a threshold for eligibility or the broad definition of HCP), the committee should have used more sophisticated models that simulate the dynamic spread of Covid‐19 in the U.S. population to quantify the potential lives saved by vaccinating members of each group.^41^ It should have also modeled the effects of different options (like the eligibility age cutoffs) on mitigating health inequities. This process could have illuminated, for example, how many lives the United States lost by vaccinating healthy, nonfrontline doctors who solely provide telehealth services instead of high‐risk adults living in communities with a high Covid‐19 burden. Notably, the ACIP employed modeling in its other work to analyze what it viewed as purely public health, rather than ethical, questions—for instance, how to incorporate vaccine side effects into recommendations.^42^

Second, although the ACIP listed three reasonable and acceptable ethical principles, the committee made no effort to rank order the principles or assign them relative weights. This approach implies that there are no trade‐offs in scarce‐resource allocation and that the chosen group during each phase was optimal according to all three principles. Yet vaccinating high‐risk adults would have saved more lives than would vaccinating just anyone loosely affiliated with a hospital. The ACIP did not acknowledge this tension, instead allowing the public to assume that HCP priority best satisfied all three principles.

One might object that these criticisms are unfair because the ACIP's membership overwhelmingly comprises clinicians and public health professionals, who do not typically design scarce‐resource allocation protocols. However, they had access to consultants as well as NASEM's expert report. The ACIP could have engaged a consultant team with domain expertise in the ethical design of scarce‐resource‐allocation protocols, or could have simply adopted NASEM's recommendations. In contrast to the membership of the NASEM committee, no author of ACIP's allocation guidance, a group that comprised both the ACIP members and consultants, held a doctorate in bioethics, philosophy, or any social science. The majority held only MDs, and only two members had doctoral degrees in nonclinical subjects (public health and health and behavioral sciences). To be sure, formal credentials are neither necessary nor sufficient to engage complex scarce‐resource‐allocation problems, but consulting with experts in the field might have helped the ACIP avoid its missteps.

The task of converting ethical principles into a vaccine‐allocation protocol was an enormous bioethical challenge. It is critical for the bioethics community to acknowledge the shortcomings of the ACIP protocol, identify the root causes of the missteps, and build a better set of empirical bioethical tools to convert principles into protocols. Lives were, and remain, on the line.
